# The Metallothionein System in *Tetrahymena thermophila* Is Iron-Inducible

**DOI:** 10.3390/toxics12100725

**Published:** 2024-10-08

**Authors:** Davide Gualandris, Davide Rotondo, Candida Lorusso, Antonietta La Terza, Antonio Calisi, Francesco Dondero

**Affiliations:** 1 Department of Science and Technological Innovation, Università degli Studi del Piemonte Orientale, Viale Michel 11, 15121 Alessandria, Italy; davide.gualandris@uniupo.it (D.G.);; 2 School of Biosciences and Veterinary Medicine, University of Camerino, 62032 Camerino, Italy; antonietta.laterza@unicam.it

**Keywords:** aquatic system, heavy metals, evolution, oxidative stress, pollution, protist, iron homeostasis

## Abstract

Metallothioneins are multifunctional proteins implicated in various cellular processes. They have been used as biomarkers of heavy metal exposure and contamination due to their intrinsic ability to bind heavy metals and their transcriptional response to both physiological and noxious metal ions such as cadmium (Cd) and mercury (Hg). In this study, we aimed to clarify the role of iron and reactive oxygen species (ROSs) in the induction of the metallothionein system (Mtt) in the ciliate protozoan *Tetrahymena thermophila*. We investigated the relative mRNA abundances of the metallothionein genes Mtt1, Mtt2/4, and Mtt5, revealing for the first time their responsiveness to iron exposure. Furthermore, by using inhibitors of superoxide dismutase (SOD) and catalase (CAT), alone or in combination with iron, we highlighted the roles of superoxide ion and endogenous hydrogen peroxide, as well as the complex interplay between the metal and ROSs. These results enhance our understanding of the metallothionein system in ciliates and suggest that ROSs may be a primary evolutionary driver for the selection of these proteins in nature.

## 1. Introduction

Metallothionein (MT) is a superfamily of proteins widely distributed among both eukaryotic [1] and prokaryotic [2] organisms. They are distinguished by their exceptional affinity for heavy metal ions and metal binding capacity, attributed primarily to a unique content of reduced cysteine residues (20–35%) in the primary structure, giving rise to metal–tetrathiolate clusters [3].

Native purified MTs typically bind Zn(II) and Cu(I) ions, particularly under physiological conditions [4]. However, their binding affinities extend to other metals in vivo, including Cd(II) and Hg(II) [5]. Furthermore, monosubstituted derivative MTs, originating from the metal-free, known as MT apo, form, can accommodate various metals such as Fe(II), an essential metal, along with Pb(II), Bi(III), Sn(II), Co(II), Ni(II), Tc(III), In(III), and Sb(III) [6]. These forms are practically artificial and do not occur in natural systems. Traditionally designated as reservoirs/buffers for physiological heavy metal ions and scavengers for toxic species like Cd, Cu and Hg [5], MTs have often been perceived to have an elusive primary function [7]. Knockout studies in fruit flies, in fact, revealed that MTs are non-essential for organismal development and survival [8]. Nevertheless, they confer significant advantages in coping with stressful situations, including exposure to heavy metals, low Zn conditions, infection, and various oxidative stress-related processes [9]. Moreover, beyond their metal ion affinity and binding capacity, MTs exhibit a substantial redox capacity, playing a pivotal role in diverse stress-response processes, such as inflammation [10], gamma rays [11] or UVB irradiation [12], and with challenges from xenobiotics triggering the formation of Reactive Oxygen Species (ROSs) [13]. Additionally, MT genes display transcriptional responsiveness to other non-physiological and physiological non-metal stimuli including glucocorticoids, and/or several polypeptide hormones [14]. Despite their interactions with various metals, no role in iron homeostasis has been attributed to MTs so far, making this an area worthy of investigation.

*Tetrahymena* spp. are ciliated protozoa that express multiple metallothionein genes, which fall into two discrete subfamilies, 7a and 7b, differing in their structural and evolutionary traits [15].

*Tetrahymena* MT genes coordinate expression with distinctive functions and respond diversely to environmental stressors. In *T. thermophila*, MTT1 (a protein encoded by the Mtt1 gene) binds and responds preferentially to Cd(II), aiding in noxious metal homeostasis and detoxification. Nearly identical, Mtt2 and Mtt4 genes encode copper MTs with the highest binding preference for Cu(I) [16]. Among its five metallothionein genes, the cadmium metallothionein Mtt5 gene is deemed essential [17].

The study of the transcriptional regulation of MT genes in Tetrahymena is noteworthy for unraveling cellular responses to metal stress and environmental adaptation. The transcriptional regulation of MT genes in *Tetrahymena* has been a subject of investigation owing to its significance in understanding cellular responses to metal stress and environmental adaptation. Two seminal studies demonstrated the robust inducibility–repressibility of the Cd-inducible Mtt1 [18] and Cu-inducible Mtt2 [19] genes in *T. thermophila*. These studies revealed that Mtt1 or Mtt2 mRNA expression is tightly regulated by cadmium or copper ion concentration, respectively, with rapid induction upon metal exposure and subsequent downregulation upon withdrawal. Moreover, both promoters were shown to be highly efficient in driving conditional expression of heterologous genes, under control of fully sub-toxic metal levels, offering a valuable tool for genetic manipulation and functional studies in *T. thermophila*, but also demonstrating definitively that Mtt1 and Mtt2 are primarily controlled by inducible promoters. Vertebrate Mt genes are transcriptionally regulated through multiple cis-acting elements known as Metal Responsive Elements (MREs) [14]. *Tetrahymena* metallothionein promoters do not appear to contain genuine MREs. Insights into the transcriptional regulation of *Tetrahymena* MT genes emerged from the work of Formigari et al. [20], showing the involvement of a proximal composite GATA cis-acting element in Cd inducibility of Mtt5 and those of Diaz et al. [15] and de Francisco et al. [21], who identified multiple examples of Metallothionein Conserved Motif 1 (MTCM1) resembling AP-1 antioxidant responsive elements, suggesting a potential role for AP-1 (bZIP) transcription factors in regulation of Mtt gene expression. AP-1 binding sites have been identified in several MT vertebrate promoters, where they are part of a complex cis-acting element setting [22,23,24].

The aim of this study is to investigate the involvement of metallothioneins in the oxidative stress response within *T. thermophila*, specifically examining how iron exposure and oxidative stress affect MT gene regulation. To achieve this objective, we employed quantitative real-time reverse transcription quantitative PCR (QPCR) in conjunction with multiplexed Taqman hydrolysis probes, allowing for precise assessment of the relative abundance levels of metallothionein mRNAs, specifically Mtt1, Mtt2/4, and Mtt5. In selecting the metallothionein genes Mtt1, Mtt2/4, and Mtt5 for analysis, we aimed to reduce complexity while ensuring a comprehensive understanding of MT gene regulation in *T. thermophila*. According to Diaz et al. [15] the coding sequences and amino acid compositions of Mtt2 and Mtt4 are nearly identical, indicating very close molecular evolution of the 7b Mtt sub-family. Due to the high nucleotide sequence similarity between them, we analyzed Mtt2 and Mtt4 together, as a set. On the other hand, Mtt3 is similar to Mtt1, with both genes appearing to have evolved from the same ancestral gene. Although Mtt5 is distinct, it shares an evolutionary origin with Mtt1 and Mtt3 [15], providing a broader perspective on MT gene regulation for the 7a Mtt sub-family protein. Our investigation focused on the gene responsiveness of metallothioneins to superoxide and hydroxyl radicals, achieved through the use of inhibitors for superoxide dismutase (SOD) and catalase (CAT), as well as the introduction of iron to initiate the Fenton [25] and Haber–Weiss [26] reactions triggering the formation of these radicals. Unexpectedly, our findings highlighted a pivotal role of iron per se in modulating gene expression within *T. thermophila* metallothionein. Understanding these interactions not only contributes to basic biological knowledge, but also has broader implications, such as insights into environmental metal toxicity and potential biomedical applications related to oxidative stress and metal homeostasis. By exploring how iron exposure and oxidative stress affect MT regulation in *T. thermophila*, we aim to shed light on the complex regulatory mechanisms of MT genes and their relevance in coping with metal-induced oxidative stress.

## 2. Materials and Methods

All chemicals were reagent grade and were purchased from Merck (Darmstadt, Germany). All dehydrated culture media powders were purchased from DIFCO™ (Franklin Lakes, NJ, USA).

### 2.1. Tetrahymena thermophila Culture and Treatments

Cultures of *T. thermophila* strain CU427 (10 mL) were grown in axenic condition in PPY culture medium (10 g/L Difco Bacto^TM^ yeast extract, 1.5 g/L Difco Bacto^TM^ proteose peptone and 30 mg/L ethylenediaminetetraacetic acid ferric sodium salt) until the late-log phase at a density of 10^6^ cells/mL, at 30 °C, 150 rpm, using aerated 50 mL polypropylene plastic Falcon^TM^ tubes. Log-exponential cultures of *T. thermophila* were treated with the following chemicals: iron(III) chloride, sodium azide, sodium diethyldithiocarbamate and hydrogen peroxide. For the selection of testing concentrations, we used No Observed Effect Concentrations (NOECs) for swimming as a proxy for survival. Briefly, late log-exponential cultures of *T. thermophila* were settled in 96 multi-well plates in full medium with the addition of increasing concentrations of each individual chemical for 24–72 h. Cultures were then gathered and assessed for swimming behavior under light microscopy at 25–50× magnification. The concentration giving rise to an unequivocal change in overall *T. thermophila* motility was referred to as the Lowest Observed Effect Concentration (LOEC). The preceding concentration was designated as the NOEC and used for subsequent experiments. The designed concentrations used in the experiments are given in Table 1.

Cadmium in the form of chloride was used at 20 μM as a positive control for Mtt1 and Mtt5 induction.

All reagents were obtained from Merck (Darmstadt, Germany) and were of ultra-pure grade, with a purity equal to or greater than 99%.

### 2.2. RNA Extraction

Total RNA was extracted with the GeneJet RNA Purification Kit (Thermo Fisher Scientific) (Waltham, MA, USA) according to the manufacturer’s instructions. This reagent system uses a silica-based membrane in a centrifuge column. Briefly, cell pellets (1–5 × 10^5^ cells) were lysed and homogenized in lysis buffer containing guanidine thiocyanate and detergents. The lysate was then mixed with ethanol and loaded onto the silica gel purification column. The lysate was centrifuged at 10,000× *g* at RT, and the flowthrough was discarded while the RNA was retained by the membrane. DNA, proteins and salts were removed from the membrane in two different washing steps using a buffered hydroalcoholic (75% ethanol) proprietary solution. Total RNA was eventually eluted under low ionic strength conditions with nuclease-free water in a variable volume of 30–50 μL.

### 2.3. cDNA Synthesis

Before reverse transcription, genomic DNA (gDNA) was removed using DNAse-I treatment [27]. Total RNA (up to 1 μg in μL nuclease-free water) was mixed with 1 U DNase-I (Thermo Fisher Scientific-(Waltham, MA, USA) in reaction buffer containing MgCl_2_, 40 U RiboLock RNase Inhibitor reagent (Thermo Fisher Scientific-Waltham, MA, USA), and incubated at 37 °C for 30 min in a final volume of 10 μL according to the manufacturer’s instructions. After DNase-I inactivation at 70 °C for 10 min, 2 μL of the reaction mixture was used for first-strand cDNA synthesis using a point-mutated RNase H-deficient M-MuLV reverse transcriptase (RevertAid H-minus, Thermo Fisher Scientifics) in the presence of 125 ng random hexamers (Invitrogen, Waltham, MA, USA), dithiothreitol, MgCl_2_ and enzyme buffer in a volume of 10 μL, according to the manufacturer’s instructions.

### 2.4. Quantification of Relative mRNA Abundances of Mtt Genes

Real-time quantitative PCR (QPCR) in combination with a Taqman^TM^ multiplex protocol was used evaluate relative mRNA abundances of the Mtt genes. This technique involves the use of hydrolysis probes with dual fluorescent labeling [28] (Table 2). The probe set designed for Mtt2 is 100% identical to Mtt4; therefore, it recognizes the joint expression of both genes. A gene probe encoding the *T. thermophila* 17S ribosomal gene was used as reference for normalizing gene expression data between samples. All primers and probes were synthetized and purified by Eurofins Genomics (Ebersberg, Germany).

The QPCR reaction (7 μL final volume) was prepared in triplicate using 2 μL of diluted cDNA (1:10 dilution from the reverse-transcription reaction) and the iQ Multiplex Power master mix (Bio-Rad Laboratories, South Granville, BC, Canda) accounting for 3 min initial denaturation (95 °C) and 45 cycles of two-step amplification: 15 sec denaturation (95 °C), 1 min annealing plus extension at 59 °C. Within each QPCR run, a standard curve was obtained for each probe set to estimate PCR efficiency (E) and linearity (R^2^) over at least 4 magnitude orders. The average E values for each target was comprised between 1.98 and 2.01, with a R^2^ higher than 0.99; therefore, for subsequent analysis of relative mRNA abundances, an E value of 2 was adopted for all targets.

### 2.5. Statistics

Real-time quantitative PCR data were analyzed in relative mode using the ΔΔCq method of Busting et al. [29], where Cq is the threshold cycle of QPCR reaction, using non-exposed *T. thermophila* culture as relative control. GraphPad Prism^®^ 9 (GraphPad Software, San Diego, CA, USA) was used to evaluate statistical significance of the Mtt mRNA relative abundances among groups by means of conventional or Welch’s ANOVA statistics in the case of heteroscedastic data. For post hoc multiple comparison, Tukey’s or Dunnett’s T3 test was performed accordingly (for details, see captions to figures). Statistical analysis was carried out on ΔCq values, i.e., Cq of GOI (Mtt1, Mtt2/4 and Mtt5) minus Cq of the housekeeping reference gene (17s rRNA).

## 3. Results

**Figure 1:**
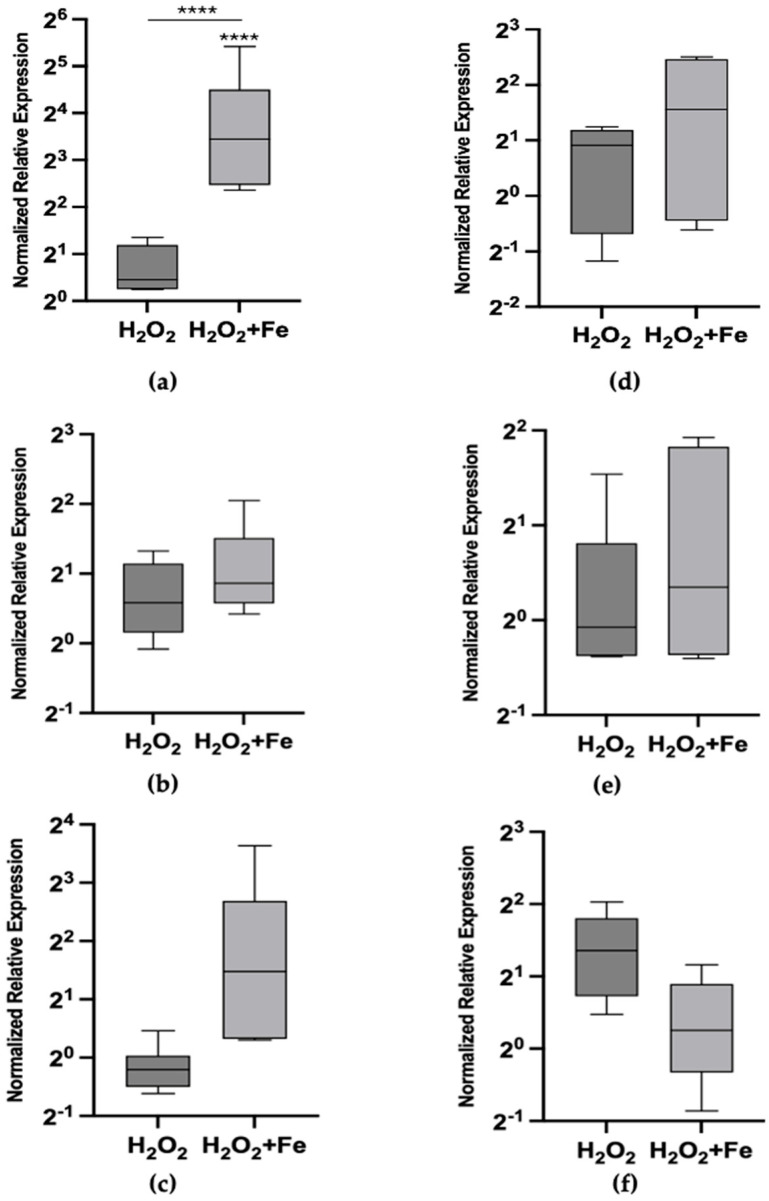
Metallothionein mRNA relative abundances (box plot) after exposure to 100 μM H_2_O_2_ and 100 μM H_2_O_2_ plus 200 μM FeCl_3_ at 24 h (**left column**) and 72 h (**right column**). (**a**) Mtt1 at 24 h, (**b**) Mtt2/4 at 24 h, (**c**) Mtt5 at 24 h, (**d**) Mtt1 at 72 h, (**e**) Mtt2/4 at 72 h and (**f**) Mtt5 at 72 h. The asterisks above each bar indicate the statistical significance of the difference in gene expression compared to control non-exposed cells. The inlet horizontal lines link the conditions that were further compared in contrasts using ANOVA. [**** *p* < 0.0001; (ANOVA, Tukey’s multiple comparison test)].

Figure 1 illustrates the effects of exogenous exposure to hydrogen peroxide and hydrogen peroxide combined with iron at 24 and 72 h. A concentration of hydrogen peroxide (Table 2) that was ineffective on its own was intentionally selected to specifically evaluate its combined effect with iron on Mtt transcriptional activation under the current experimental conditions. When combined with iron, Mtt1 showed a statistically significant activation (*p* < 0.0001), resulting in an 15.7-fold increase in expression, while Mtt5 and Mtt2/4 exhibited lower, non-significant increases in expression at 24 h (*p* > 0.1) (Figure 1a–c). No statistically significant effects were observed at 72 h, as the fold changes in expression were not different from control levels. This may indicate an adaptive response, where cells normalize Mtt expression after prolonged exposure.

**Figure 2:**
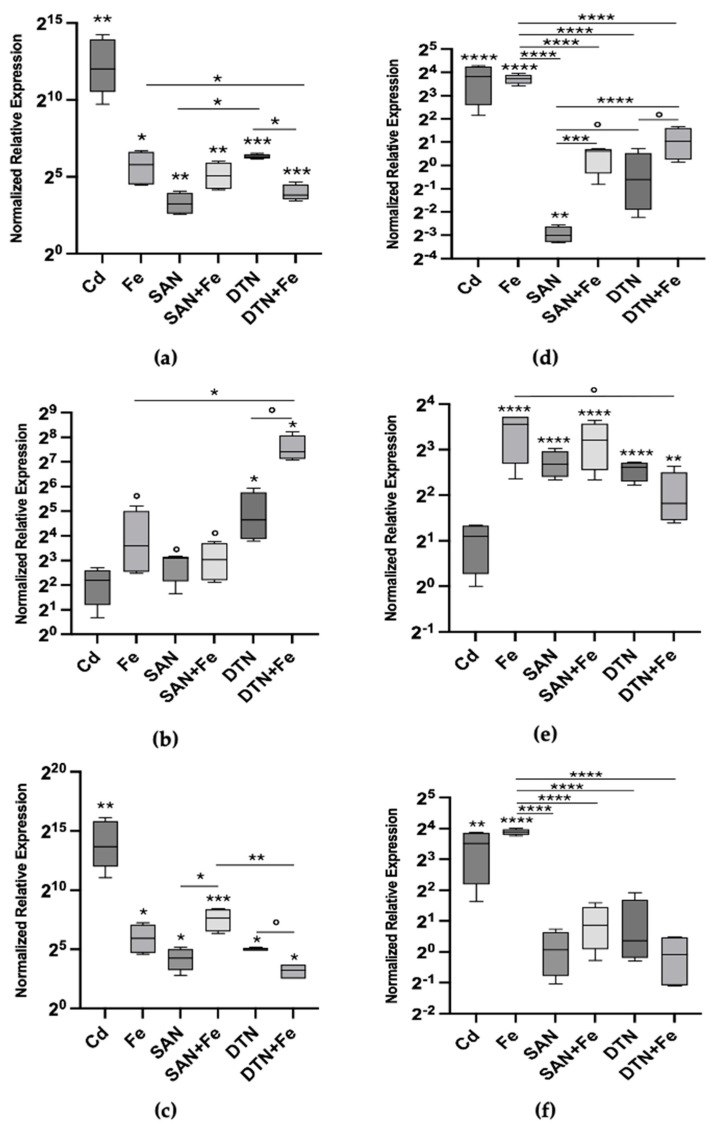
Metallothionein mRNA relative abundances (box plots) after 24 h (**left column**) and 72 h (**right column**) exposure to 20 μM CdCl_2_, 200 μM FeCl_3_, 200 μM sodium azide (SAN), sodium azide plus iron (SAN + Fe), 100 μM sodium diethyldithiocarbamate (DTN) and sodium diethyldithiocarbamate plus FeCl_3_; (**a**) Mtt1 24 h; (**b**) Mtt2/4 24 h; (**c**) Mtt5 24 h; (**d**) Mtt1 72 h; (**e**) Mtt2/4 72 h and (**f**) Mtt5 72 h. The asterisks above each bar indicate the statistical significance of the difference in gene expression compared to control non-exposed cells. The inlet horizontal lines link the conditions that were further compared in contrasts using ANOVA. Significance levels: [**** *p* < 0.0001; *** *p* < 0.001; ** *p* < 0.01; * *p* < 0.05; ° *p* < 0.1 ((**a**–**c**) Welch’s ANOVA, Dunnett’s T3 multiple comparison test; (**d**–**f**) ANOVA, Tukey’s multiple comparison test)]. Cadmium was used as a positive control, and was not included in contrasts.

The relative expression levels of Mtt1, Mtt2/4, and Mtt5 were measured after 24 h of exposure to various stressors (Table 2), including Cd(II) as a positive control, Fe(III), sodium azide, sodium diethyldithiocarbamate, and combinations of each inhibitor with iron (Figure 2a–c). Cd(II) exposure resulted in the highest fold change in gene expression, with a substantial 4152-fold increase in Mtt1 expression (*p* < 0.01). Iron induced Mtt1 expression to a lesser extent, showing a 55.7-fold increase (*p* < 0.05). The inhibitors sodium azide and sodium diethyldithiocarbamate also caused statistically significant increases in Mtt1 expression, with fold changes of 9.38 and 79.33 (Figure 2a).

Interestingly, the combination of sodium azide and iron resulted in a higher Mtt1 expression than sodium azide alone, with a fold change similar to that of iron treatment, suggesting that the effect is primarily driven by iron. In contrast, diethyldithiocarbamate combined with iron showed a statistically significant lower expression level than diethyldithiocarbamate alone (*p* < 0.05), indicating an antagonistic interaction between the inhibitor and iron in modulating Mtt1 expression (Figure 2a).

For Mtt2/4 (Figure 2b), iron exposure alone resulted in an increase in gene expression at 24 h, despite being only marginally statistically significant (*p* < 0.1), leading to a 12.12-fold increase compared to the control. In contrast, cadmium exposure did not result in a significant change in Mtt2/4 expression (*p* > 0.1), with median values lower than those induced by iron. This indicates that Mtt2/4 is more responsive to iron than to cadmium, which contrasts with the response pattern observed in Mtt1 and is in line with the overall feature of the Cu inducible gene of the 7a Mtt sub-family.

Sodium azide and diethyldithiocarbamate also elicited increases in Mtt2/4 expression. Sodium azide caused a 8.48-fold increase, while diethyldithiocarbamate induced a 25.02-fold increase compared to control levels. When combined with iron, sodium azide showed no additional effect on Mtt2/4 expression beyond that induced by iron alone. However, diethyldithiocarbamate plus iron caused a sharp increase, yielding the highest fold change observed for this isotype—a 170.09-fold increase. This suggests a possible additive or synergistic effect between diethyldithiocarbamate and iron in inducing Mtt2/4 expression.

Similarly, for Mtt5 (Figure 2c), cadmium exposure resulted in the highest expression, with a substantial and highly statistically significant 13,079-fold increase over control levels (*p* < 0.01). The pattern of Mtt5 expression was very similar to that of Mtt1. Iron exposure induced a significant increase in Mtt5 expression, showing a 60.75-fold increase (*p* < 0.05). When co-exposed with inhibitors, the effects on Mtt5 expression mirrored those observed with Mtt1. Remarkably, the difference between sodium azide alone (19.04-fold increase) and sodium azide plus iron (194.67-fold increase) was statistically significant (*p* < 0.05), likely due to lower variability in the measured values of normalized fold change. This indicates an additive effect of iron in the presence of sodium azide on Mtt5 expression.

After 72 h of exposure, we assessed the expression patterns of Mtt1 (Figure 2d), Mtt2/4 (Figure 2e), and Mtt5 (Figure 2f) under the same conditions as the 24 h exposure. For Mtt1, the relative mRNA abundance declined compared to the 24 h levels, reaching new steady-state levels across most treatments. Specifically, cadmium-induced Mtt1 expression decreased from a 4152-fold increase at 24 h to a 14.14-fold increase over control at 72 h (*p* < 0.0001). However, iron-treated samples maintained substantial Mtt1 expression, with a 13.17-fold increase over control (*p* < 0.0001), which was significantly higher than any other condition analyzed at 72 h (*p* < 0.0001).

Treatment with sodium azide resulted in Mtt1 expression levels significantly below control levels, indicating repression of Mtt1 transcription. Sodium azide led to a 0.12-fold decrease relative to the control (*p* < 0.01), while diethyldithiocarbamate resulted in no statistically significant change compared to the control. When combined with iron, the expression levels of Mtt1 in the sodium-azide-plus-iron and diethyldithiocarbamate-plus-iron treatments were significantly higher than those of the individual inhibitors alone, but lower than that of iron alone. At 72 h, the combination treatments of sodium-azide-plus-iron and diethyldithiocarbamate-plus-iron resulted in Mtt1 expression levels that were not significantly different from the control group. Although these levels were similar to control, they were significantly higher than those observed with the inhibitors alone, but significantly lower than the 15-fold increase observed with iron alone (Figure 2d).

At 72 h, iron exposure continued to induce substantial Mtt2/4 expression (Figure 2e), resulting in an 11.76-fold increase over control levels (*p* < 0.0001). Sodium azide and diethyldithiocarbamate treatments also maintained elevated Mtt2/4 expression, with fold changes of 6.39-fold and 6.10-fold, respectively (*p* < 0.0001). Combining sodium azide with iron did not produce any important additional effect on Mtt2/4 expression, yielding a fold change of 9.23-fold over control (*p* < 0.0001), similar to iron alone. However, the combination of diethyldithiocarbamate and iron resulted in a statistically significant reduction in Mtt2/4 expression, to a 3.52-fold increase over control (*p* < 0.01), which was lower than both the 24 h level and the 72 h iron level. These findings suggest an interaction between iron and diethyldithiocarbamate, potentially indicating an adaptive response of Mtt2/4 under these conditions.

The expression pattern of Mtt5 after 72 h mirrored that of Mtt1. Cadmium exposure continued to induce statistically significant Mtt5 expression, with an 11.45-fold increase over control levels (*p* < 0.01). Iron treatment resulted in a 14.81-fold increase in Mtt5 expression (*p* < 0.0001). The treatments with sodium azide, diethyldithiocarbamate and their combinations with iron showed no significant effect on Mtt5 expression (*p* > 0.1). This indicates that the inhibitors attenuated the iron-induced expression of Mtt5. The rapid adjustment of Mtt5 expression levels might reflect a more dynamic response mechanism to prolonged exposure under these conditions.

**Figure 3:**
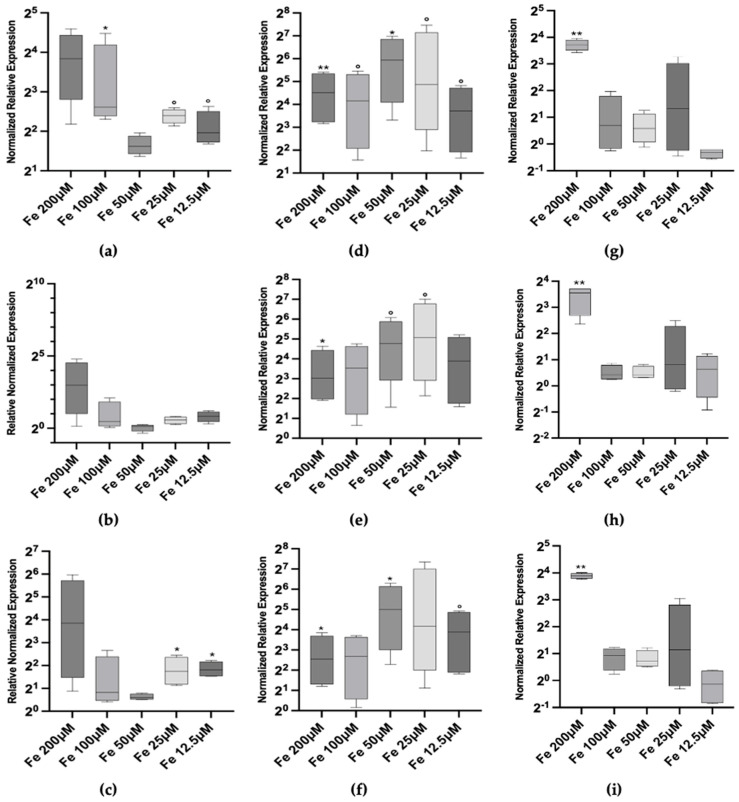
Metallothionein mRNA relative abundances (box plots) after 2 h (**left column**), 24 h (**center column**) and 72 h (**right column**) exposure to iron. (**a**) Mtt1 2 h; (**b**) Mtt2/4 2 h; (**c**) Mtt5 2 h; (**d**) Mtt1 24 h; (**e**) Mtt2/4 24 h; (**f**) Mtt5 24 h; (**g**) Mtt1 72 h; (**h**) Mtt2/4 72 h and (**i**) Mtt5 72 h. Significance levels: the asterisks above each bar indicate the statistical significance of the difference in gene expression compared to control non-exposed cells [** *p* < 0.001; * *p* < 0.05; ° *p* < 0.1 (Welch’s ANOVA, Dunnett’s T3 multiple comparison test vs. non-exposed reference control)].

Given the observed induction of Mtt genes by iron, we focused on investigating iron as a potential novel inducer of Mtt expression, examining dose-dependent effects ranging from 12.5 µM to 200 µM and kinetic responses over time periods from 2 h to 72 h (Figure 3).

At the 2 h time-point, we observed a positive transcriptional response of Mtt1 and Mtt5 at both low and high iron concentrations. Specifically, Mtt1 expression increased significantly at 12.5 and 25 µM (*p* < 0.1) by 3.89- and 5.28-fold, compared to control, respectively. At 100 and 200 µM, the median expression level is higher (5.6- and 8-fold) but only the former is statistically significant (*p* < 0.05) (Figure 3a). Similarly, Mtt5 expression showed a 3.40-fold increase at 12.5 and 25 µM (*p* < 0.05) and a 14.47-fold increase at 200 (Figure 3c). However, high variability in the data led to larger uncertainty levels in this condition (*p* > 0.1). In contrast, Mtt2/4 exhibited only a marginal activation at the highest iron concentration of 200 µM, with a median 7.92-fold increase over control (*p* > 0.1), indicating a less pronounced response at the early time-point (Figure 3b).

At 24 h, the transcriptional responses of all three Mtt genes peaked. Mtt1 expression averaged 32 times higher than the control, with a maximum of 61.67-fold increase at 50 µM iron (*p* < 0.05) (Figure 3d). Mtt5 expression mirrored this pattern, with a 32.11-fold increase at 50 µM (*p* < 0.05) (Figure 3f). Mtt2/4 also demonstrated statistically significant induction (despite *p* < 0.1) at all concentrations, with a peak at 25 and 50 µM iron and with a fold increase, respectively, of 33.7 and 27.36 (*p* < 0.1) (Figure 3e).

At 72 h, the expression levels of Mtt1, Mtt2/4 and Mtt5 did not return to baseline at the highest iron concentration (200 µM), settling at levels of 13.17-, 11.66- and 14.8-fold increase in the three cases, and suggesting a sustained response to high iron levels (Figure 3g–i).

Throughout the experiment, no clear dose–response pattern was observed for any of the Mtt genes. The lack of a linear dose–dependent effect may suggest that *T. thermophila* regulates intracellular iron concentrations tightly, possibly involving feedback mechanisms that modulate Mtt gene expression. The adaptive response observed over time, with peak expression at 24 h followed by a decline, indicates that the cells adjust their gene expression in response to prolonged iron exposure, and that a possible condition of iron repletion at the highest concentration still needs compensation.

These findings imply that Mtt genes may play a role in iron homeostasis in *T. thermophila*, contributing to the regulation of intracellular iron levels and protection against iron-induced oxidative stress.

Given the observed induction of Mtt genes by iron, we focused on investigating iron as a potential novel inducer of Mtt expression, examining dose–dependent effects ranging from 12.5 μM to 200 μM and kinetic responses over time periods from 2 h to 72 h (Figure 3).

At the 2 h time-point, we observed a positive transcriptional response of Mtt1 and Mtt5 at both low and high iron concentrations. Specifically, Mtt1 expression increased significantly at 12.5 μM and 25 μM, 3.89-fold and 5.28-fold compared to control, respectively, (*p* < 0.1 and *p* < 0.05). At 100 μM and 200 μM, the median expression levels were higher (approximately 6.1-fold and 14.34-fold, respectively), with the increase at 100 μM being statistically significant (*p* < 0.05) (Figure 3a). Similarly, Mtt5 expression showed a 3.5-fold increase at 12.5 μM and 25 μM (*p* < 0.05) and a 14.47-fold increase at 200 μM (Figure 3c). However, high variability in the data led to larger uncertainty levels at the highest concentration (*p* > 0.1). In contrast, Mtt2/4 exhibited only a marginal activation at the highest iron concentration of 200 μM, with a median 7.92-fold increase over control (*p* > 0.1), indicating a less pronounced response at the early time-point (Figure 3b).

At 24 h, the transcriptional responses of all three Mtt genes peaked. Mtt1 expression averaged 39 times higher than the control, with a maximum 61.64-fold increase at 50 μM iron (*p* < 0.05) (Figure 3d). Mtt5 expression mirrored this pattern, with a 39-fold increase at 50 μM (*p* < 0.05) (Figure 3f). Mtt2/4 also demonstrated statistically significant induction at almost all concentrations, with peaks at 25 μM and 50 μM iron, showing fold increases of 27.3-fold and 33.7-fold, respectively (*p* < 0.1) (Figure 3e).

At 72 h, the expression levels of Mtt1, Mtt2/4, and Mtt5 did not return to baseline at the highest iron concentration (200 μM), settling at levels of almost 13.24-fold increase in all three cases (Figure 3g–i). This suggests a sustained response to high iron levels. However, at lower concentrations, the expression levels decreased towards baseline, indicating an adaptive response over time.

Throughout the experiment, no clear dose–response pattern was observed for any of the Mtt genes. The lack of a linear dose–dependent effect may suggest that *T. thermophila* tightly regulates intracellular iron concentrations, possibly involving feedback mechanisms that modulate Mtt gene expression. The adaptive response observed over time, with peak expression at 24 h followed by a decline, indicates that the cells adjust their gene expression in response to prolonged iron exposure and possibly reach a condition of iron repletion at lower concentrations. However, at the highest concentration, the sustained elevated Mtt expression suggests that the cells still need compensation and have not fully adapted. These findings imply that Mtt genes may play a role in iron homeostasis in *T. thermophila*, contributing to the regulation of intracellular iron levels and protection against iron-induced oxidative stress. The expression patterns among the Mtt genes suggest similar roles or regulatory mechanisms in response to iron, with Mtt1 and Mtt5 showing more consistent patterns with each other.

## 4. Discussion

Our initial hypothesis centered around confirming Tetrahymena Mtt’s involvement in ROS scavenging activity. Consequently, hydrogen peroxide and a combination of hydrogen peroxide with iron were used as exogenous sources of ROSs. Furthermore, inhibitors targeting key antioxidant enzymes were employed, specifically sodium azide [30] and sodium diethyldithiocarbamate [31], to inhibit catalase (CAT) and superoxide dismutase (SOD) activities, respectively, thereby promoting the intracellular formation of ROSs. Previous studies have demonstrated the efficacy of this strategy to raise levels of hydrogen peroxide [32] or superoxide ion [33] using micromolar levels of the same inhibitors employed in this work. To intensify this effect, iron was included as an additional treatment with the aim of stimulating the Fenton [25] and/or Haber–Weiss [26] reactions known to generate hydroxyl and/or hydroperoxyl radicals from hydrogen peroxide (H_2_O_2_) or superoxide (O_2_^−^), thus gathering and assessing the transcriptional modulation of the Mtt genes using QPCR quantifying relative abundances of *T. thermophila* metallothionein mRNAs (Mtt1, Mtt2/4, Mtt5).

Sodium azide was used due to its ability to inhibit catalase [30]. This enzyme is responsible for the conversion of hydrogen peroxide into molecular oxygen and water [34]. This is a fundamental defense mechanism that all organisms use to detoxify endogenous H_2_O_2_, and if this molecule is not taken care of, it reacts and degrade proteins, nucleic acids, and lipids. Sodium diethyldithiocarbamate was used because it is a known inhibitor of Super Oxide Dismutase (SOD) [31]. This enzyme is responsible for the dismutation reaction of the superoxide anion (O_2_^−^) into two different compounds (H_2_O_2_ and O_2_) [35]. SOD is a key enzyme in the cell’s defense against superoxide anion, a ROS which causes the inactivation of iron/sulfur clusters present in many enzymes and proteins that play a key role in metabolic processes.

In addition to exacerbating the intracellular oxidation, two inhibitors with iron were paired; therefore, three new treatments were obtained, i.e., sodium azide plus iron, sodium diethyldithiocarbamate plus iron and H_2_O_2_ plus iron. Iron, in fact, must be tightly regulated intracellularly, as it is the starting point of several reactions that create oxidative stress (sensu the Fenton [25] or Haber–Weiss reactions [26]). Previous data based on comparative QPCR analyses and protein-DNA interaction experiments supported the involvement of AP-1 transcription factors in mediating MT gene expression responses to metal stress in *T. thermophila* [21]. The latter finding is quite relevant in the context of the gene expression results of our study, since AP-1 transcription factors are responsible for the transcriptional control of oxidative stress-related genes through the Antioxidant Response genetic Elements (AREs). In mammalian cells, these factors can bind to specific cis-acting elements rendering numerous cellular processes, including cell proliferation, apoptosis, and development, but also triggering response to stress induced by exposure to UV or cytotoxic agents (e.g., cycloheximide, 4-nitroquinoline), iron-chelating complexes, oxidizing agents and several heavy metals including iron [36,37]. AP-1 transcription factors seem to be universal mediators, as they are also relevant for abiotic stress response in yeast [38] and plants [39]; moreover, they have already been mapped in MT promoters of vertebrates [22,23,24]. In addition to transcription factors, epigenetic mechanisms such as microRNAs (miRNAs) have been implicated in post-transcriptional regulation of genes involved in metal-stress responses. Amaro et al. [40] isolated and characterized miRNAs involved in the post-transcriptional regulation of transcripts linked to the response to cadmium stress in *T. thermophila*. These studies highlight the regulatory complexity underlying the cellular response to metals in modulating gene expression in *Tetrahymena*, even in a relatively simpler model organism. They also emphasize the inevitable interplay between heavy-metal and oxidative stress responses, as has been previously described in vertebrates.

The activation of *T. thermophila* metallothionein genes by oxidants such as hydrogen peroxide is well documented in the literature [41,42]. Our study, however, provides new insights into the response of metallothioneins to abiotic stimuli. Based on the hypothesis that the repression of catalase and SOD enzyme activities could increase the levels of H_2_O_2_ and superoxide radicals, a pioneering investigation into the influence of iron on metallothionein gene expression was carried out, focusing specifically on the *T. thermophila* Mtt system.

Our work represents a substantial advancement in the understanding of the molecular regulation of metallothionein genes. By targeting key antioxidant enzymes with specific inhibitors and co-exposing protozoa to iron, the study aimed to simulate endogenous oxidative stress conditions conducive to MT gene induction. This innovative approach allowed us to explore the direct impact of reactive oxygen species (ROSs) on MT gene expression. A key question is whether the two inhibitors could activate Mtt gene transcription, and this study provides clear evidence supporting that. This activation may result from either a direct effect of increased endogenous ROS levels or from indirect effects of ROSs on intracellular metal levels (and their oxidation state).

However, it is important to consider that diethyldithiocarbamate is not only a SOD inhibitor but also a potent chelating agent for metal ions, including iron [43]. Diethyldithiocarbamate can form stable iron(II/III)–dithiocarbamate complexes, which may alter the bioavailability of iron within the cell [44]. The formation of such complexes could sequester free iron ions, potentially decreasing their availability to enter the cell and modulate Mtt gene expression. Additionally, iron–diethyldithiocarbamate complexes have been reported to generate reactive oxygen species through redox cycling, exacerbating oxidative stress [45]. This dual role of diethyldithiocarbamate could complicate the interpretation of our results, as the observed effects might be influenced by both SOD inhibition and iron chelation or redox activity of the complexes. The antagonistic effect observed on Mtt1/Mtt5 expression when diethyldithiocarbamate is combined with iron may, therefore, result from decreased availability of free iron ions due to complexation, or from increased oxidative stress due to redox cycling of the iron–diethyldithiocarbamate complexes. This highlights the need to consider the chemical interactions between experimental compounds and their potential impact on cellular responses.

Another remarkable finding is that iron itself effectively modulated Mtt gene expression. The combination of iron with sodium azide or hydrogen peroxide never exceeded the effects of iron alone, suggesting non-significant interactions, at least by 24 h (Figure 2a–c). Indeed, at 72 h (Figure 2d–f), the effects of iron combined with sodium azide were significantly lower than those of iron alone for Mtt1 and Mtt5, but not for Mtt2/4. This finding suggests differential regulation between the 7a and 7b groups of Mtt genes, indicating that Mtt2/4 might have a unique regulatory mechanism compared to Mtt1 and Mtt5. Comprehensive mechanistic explanations for this behavior cannot be determined from gene expression data alone, necessitating further studies.

It is important to note that our inability to distinguish between Mtt2 and Mtt4 expression due to their high sequence similarity represents an important limitation of this study. This lack of distinction could mask important differences in their regulation and function, potentially leading to oversimplified interpretations of their roles. Future studies employing gene-specific probes in the untranslated region, RNA sequencing, or other molecular techniques capable of discriminating between these closely related genes are necessary to elucidate their individual contributions to MT regulation in response to iron and oxidative stress.

Regarding the combined effects of exogenous hydrogen peroxide and iron at 72 h, a possible explanation can be drawn from an analogy with *S. cerevisiae*, where iron uptake involves a membrane-bound ferrireductase to reduce Fe(III) to Fe(II) and a ferrous transporter (Ftr1p) to import Fe(II) into the cell [46,47]. Exogenous hydrogen peroxide may reduce Fe(III) to Fe(II), interfering with iron’s availability to enter the cell and modulate Mtt gene expression. Simultaneously, the interaction between endogenously formed hydrogen peroxide, due to catalase inhibition by sodium azide, might alter the oxidation state of iron over time, affecting its ability to regulate Mtt1 and Mtt5 expression. This suggests that the observed effects at 72 h are the result of a time-dependent process that requires further investigation to fully understand the underlying mechanisms.

The delayed response of Mtt1 and Mtt5 when exposed to iron combined with sodium azide raises interesting questions about long-term regulation. Without further mechanistic insights, this remains an open area of inquiry. Future studies should focus on detailed time-course analyses, potentially examining gene expression at multiple time-points to capture the dynamics of MT regulation over time. Additionally, investigating the involvement of specific signaling pathways or transcription factors that might be modulated over prolonged exposure could provide deeper insights into the regulatory mechanisms at play.

Furthermore, it is important to acknowledge that at the high iron concentrations used in our experiments (100–200 µM), there is a potential for initiating ferroptosis—a form of regulated cell death dependent on iron and characterized by the accumulation of lipid peroxides [48]. Ferroptosis is associated with oxidative stress and can influence gene expression patterns [49]. While ferroptosis has been extensively studied in mammalian cells, its occurrence in unicellular organisms like *T. thermophila* is less understood. If ferroptosis or ferroptosis-like pathways are activated in Tetrahymena at high iron levels, this could influence MT expression as part of a protective response against iron-induced oxidative stress. The lack of clear concentration dependence in Mtt gene expression may reflect the complex interplay between iron accumulation, oxidative stress, and cell death pathways. Further studies are necessary to determine whether ferroptosis contributes to the observed effects and how it impacts MT gene regulation in Tetrahymena.

A consistent pattern of iron inducibility was observed across all Mtt genes (Figure 3), characterized by an adaptive response that lowered its intensity over time, except at high, non-permissive levels (200 µM), where gene expression remained substantial throughout. Unlike Cd and Cu exposures, iron did not show dose dependency in Mtt gene transcriptional activity, likely due to the tight regulation of free iron intracellular concentrations [50].

Another potential factor influencing MT gene expression in response to iron is the Hypoxia-Inducible Factor (HIF) pathway. Under normal conditions, HIF-α subunits are hydroxylated by prolyl hydroxylase domain enzymes (PHDs), which require iron (Fe^2^⁺) and oxygen to function. This hydroxylation marks HIF-α for degradation. High intracellular iron levels generally support PHD activity; however, excessive iron can lead to increased production of reactive oxygen species (ROSs) through Fenton reactions [51]. Elevated ROS levels can inhibit PHD activity indirectly by oxidizing the iron in the active site or modifying the enzymes [48,49]. This oxidative inhibition of PHDs leads to HIF-α stabilization, even under normoxic conditions. Therefore, the high intracellular iron levels in our experiments could potentially mimic hypoxia by promoting oxidative stress, thereby activating HIF pathways and influencing MT gene expression. While the HIF pathway is well characterized in mammals, its presence and role in unicellular organisms like *T. thermophila* are not fully established. However, evidence suggests that HIF-like mechanisms or other redox-sensitive transcription factors may exist in protozoa [43]. The induction of MT genes at high iron concentrations observed in our study might be mediated by such pathways, and this possibility warrants further investigation to elucidate the molecular mechanisms involved.

Our results open up the intriguing hypothesis that the Mtt system participates in iron homeostasis. Despite a native Fe-bound metallothionein (MT) never being isolated so far, it is interesting to note that hepcidin binds one Fe(III) ion through a tetrahedral thiolate cluster [52], similar to how metallothionein binds multiple Cd/Cu atoms. Hepcidin, a liver hormone, is the overarching governor of iron homeostasis in the plasma of mammals [53]. Very little is known about iron homeostasis in ciliates; therefore, further research is essential to understand the potential roles and mechanisms involved.

However, the evidence supporting the involvement of MTs in iron homeostasis in *T. thermophila* is not robust enough to fully endorse this claim. While our study demonstrates iron inducibility of Mtt genes, direct evidence of metallothioneins regulating intracellular iron levels is lacking. Future experiments should aim to provide more definitive evidence, such as assessing the iron-binding capacity of MT proteins through biochemical assays or investigating protein-level interactions with iron. Additionally, exploring whether MTs can influence iron storage, transport, or utilization within the cell would offer valuable insights into their potential role in iron metabolism.

Examining the effects of diethyldithiocarbamate, a superoxide dismutase (SOD) inhibitor, combined with iron (Figure 2), observes a discordant pattern between the Mtt1/Mtt5 pair and Mtt2/4. Mtt1 and Mtt5 are phylogenetically related, with consistently aligned transcriptional patterns in our study. In contrast, diethyldithiocarbamate plus iron exhibited an antagonistic effect on Mtt1/Mtt5 expression while having an additive, if not cooperative, effect on Mtt2/4. This suggests that in co-exposures, iron alone can account for the transcriptional activation of Mtt1/Mtt5, but not Mtt2/4. The response observed indicates that either iron, or superoxide ions whose levels are elevated by SOD inhibition, have distinct modes of action on the Mtt2/4 promoter. Alternatively, this interaction may generate new effector(s), possibly through the Haber–Weiss reaction, which are more effective in activating Mtt2/4; i.e., the hydroperoxyl (HO_2_•) and hydroxyl radical (OH•) are possible candidates.

While we speculate about the differential regulation of Mtt genes, the mechanistic explanations remain largely hypothetical. The lack of direct evidence for the involvement of specific transcription factors like AP-1 or potential redox sensors leaves this aspect of the discussion speculative. To strengthen our conclusions, future studies should focus on identifying and characterizing the transcription factors involved in MT gene regulation in response to iron and oxidative stress. Techniques such as chromatin immunoprecipitation (ChIP), electrophoretic mobility shift assays (EMSAs), or reporter gene assays could be employed to validate the role of these transcription factors or redox-sensitive elements.

Our findings highlight the complex interplay between iron, ROS, and the regulatory mechanisms governing MT gene expression. The evolution of metallothionein and heavy-metal-inducible gene transcription systems is a fascinating topic that intertwines the roles of oxidative stress and heavy metal detoxification. While it is clear that this system is primarily known for its role in metal ion homeostasis and detoxification, we cannot exclude the possibility that oxidative stress response has driven their evolution. This study underscores the importance of considering both direct and indirect effects of oxidative stress and metal ions in understanding the molecular regulation of MT genes. Considering the previously described effects of cadmium and copper, it is evident that ROS alone cannot be regarded as the primary factor for MT gene activation. Metals can promptly induce and repress MT gene transcription, suggesting the involvement of a metal-sensitive transcription factor analogue to the MTF-1 [54] found in vertebrate systems. Rather than a zinc finger-like MTF-1, we speculate the existence of a redox-sensitive transcription factor in *T. thermophila*, akin to the *E. coli* SoxR protein [55], or the *S. cerevisiae* Yap5 [56], which are indeed Fe-S (iron–sulfur) proteins, known for their essential role in response to oxidative stress. The latter, in particular, is known for regulating iron storage and is a basic leucin zipper (bZIP) protein, the same family of transcription factors putatively identified by de Francisco [21] in Mtt promoters. This hypothetical factor may function as a redox sensor that recognizes superoxide and/or other ROSs, while also being influenced by metal ions with high affinity for sulfur.

The comparison of iron with other metals like cadmium and copper provides additional context for understanding MT gene regulation. Both cadmium and copper have been shown to induce MT gene expression, potentially through similar or distinct regulatory pathways. Investigating whether the same transcription factors or signaling mechanisms are activated by different metals could highlight the specificity or universality of the MT response. Comparative studies examining the promoter regions of Mtt genes for conserved cis-acting elements responsive to various metals, as well as functional analyses of their regulatory sequences, would enhance our understanding of how *T. thermophila* coordinates its response to different metal exposures.

An important question arises: what has driven the evolution of metallothionein and heavy metal-inducible gene transcription systems? Can we truly exclude the possibility that oxidative-stress-response mechanisms could have driven the evolution of inducible systems that incidentally respond to heavy metals? The dual functionality of MT in both metal detoxification and oxidative stress response suggests an evolutionary advantage in environments where organisms are exposed to both metals and oxidative stress. However, oxidative stress is a common challenge faced by aerobic organisms, leading to the production of ROSs, which are highly reactive and can cause cellular damage. The evolution of MT may have been driven by the need to mitigate this damage. Additionally, MT, by binding metal ions, can reduce the catalytic activity of metals like copper or iron, which participate in Fenton and Haber–Weiss reactions, generating harmful hydroxyl radicals. The ability of MT to scavenge ROSs directly provides an additional layer of protection, and binding heavy metals may not represent the primary function of MT but rather a structural constraint to keep cysteine residues in a reduced state, acquiring redox potential and scavenging activity against oxidative radicals, as already shown [11]. The incidental responsiveness to heavy metals could be an advantageous byproduct of this primary function. In mammals, it has been shown that metals like cadmium and copper, as well as ROSs or disulfides like oxidized glutathione, can displace zinc in MT, leading to changes in MT conformation and, consequently, increased gene transcription through the activation of the proper transcription factors [57].

In summary, while oxidative stress plays a significant role in Mtt gene activation, the primary regulatory mechanism likely involves a complex interplay between ROSs and metal-responsive elements, mediated by specialized transcription factors that respond to both oxidative and metal-induced signals. The evolution of MT and its transcription system may have been driven by the need to protect against oxidative damage, with the incidental benefit of metal detoxification.

The Mtt system’s response to iron suggests potential involvement in iron metabolism. Mtt2/4 promptly responded to the interactive effects of superoxide and iron and exhibited prolonged expression in response to elevated endogenous hydrogen peroxide and superoxide levels. Given its earlier molecular evolution [15] and responsiveness to Cu [16], we speculate that Mtt2/4 might better represent the antioxidant function of the ancestral MT gene, while the 7a group may have evolved later, acquiring specialized functions related to heavy metal homeostasis, including iron. Indeed, Mtt5, which is an essential gene [17], appears to be a strong candidate for specifically controlling essential metals such as iron. However, further research is needed to validate this hypothesis, particularly considering the limitations of this study. Identifying the regulatory elements and factors involved will help better understand the evolutionary pressures that shaped these multifunctional proteins.

The ciliate *T. thermophila* represents an appropriate experimental system for this purpose.

## 5. Conclusions

In conclusion, we have characterized the transcriptional response of *T. thermophila* metallothionein isoforms (Mtt1, Mtt2/4, and Mtt5) to iron and reactive oxygen species (ROSs) using catalase and superoxide dismutase inhibitors. This study highlights the differential responses of the 7a and 7b Mtt groups, revealing the complex interplay between these factors and their regulatory mechanisms. Our findings underscore the importance of further research to understand the specific roles of these isoforms in metal homeostasis —including iron—and oxidative stress response.

## Figures and Tables

**Table 1:** Substances tested for the effects on Mtt gene expression.

Treatment	NOEC
Sodium azide (SAN)	200 μM
Sodium diethyldithiocarbamate (DTN)	100 μM
Cadmium chloride (Cd)	20 μM
Ferric chloride (Fe)	200 μM
Hydrogen peroxide (H_2_O_2_)	100 μM

**Table 2:** Primers and probes used for QPCR. NCBI GeneID is given in the name of the oligonucleotide/probe. All sequences are given in the 5′-3′ direction. Abbreviations. FAM, 6-carboxyfluorescein; Hex, hexachlorofluorescein; RED, Texas Red^TM^; BHQ1, Black Hole Quencer 1; BHQ2, Black Hole Quencer 2. F, Forward primer; R, Reverse primer.

NCBI ID	Sequence (5′-3′)	Oligo Type
**AF537326-Mtt1**	FAM-TGC TGC ACA GAC CCT AAC AGC GGA-BHQ1	Dual Labeled Probes
**AY204351-Mtt2**	HEX-TGC TTG CAA TTG CAA ACC TTG CGA-BHQ1	Dual Labeled Probes
**AY884209-Mtt5**	RED-CTC ACT AGG GCA GCA GCA CCA CTT-BHQ2	Dual Labeled Probes
**M10932-17s**	CY5-CCG CAG GTT CCA CTT CTG GTG TGC-BHQ2	Dual Labeled Probes
**AF537326-Mtt1F**	TGC TGT GGT GTA AAT GCT AAG CC	Forward PCR Primer
**AF537326-Mtt1R**	TCC TGT ACC AGT GCA ACA TCC CT	Reverse PCR Primer
**AY204351-Mtt2/4F**	ACC TCT CTG CAA ATG TGG AAC TAC	Forward PCR Primer
**AY204351-Mtt2/4R**	CCA CAG CTT TCA GTA ACA CCA CAT	Reverse PCR Primer
**AY884209-Mtt5F**	TCT GGT GAA AGC ACT AAA ATT TGT	Forward PCR Primer
**AY884209-Mtt5R**	ATC AGA ATT GCA GCA ATT TTG GG	Reverse PCR Primer
**M10932-17sF**	CGA TCA GAT ACC GTC GTA GTC TTA	Forward PCR Primer
**M10932-17sR**	GAG TTT CCC CGT GTT GAG TCA	Reverse PCR Primer

## Data Availability

The original contributions presented in the study are included in the article; further inquiries can be directed to the corresponding author.
